# Cost utility and cost-effectiveness of the APPLE-Tree programme: Active Prevention in People at risk of dementia through Lifestyle, bEhaviour change and Technology to build REsiliEnce: economic evaluation embedded within a randomised controlled trial

**DOI:** 10.1093/ageing/afag176

**Published:** 2026-06-22

**Authors:** Harriet Demnitz-King, Claudia Cooper, Michaela Poppe, Jessica Budgett, Sweedal Alberts, Larisa Duffy, Mariam Adeleke, Julie Barber, Elisa Aguirre, Henry Brodaty, Alexandra Burton, Paul Higgs, Jonathan Huntley, Helen C Kales, Iain A Lang, Natalie L Marchant, Sarah Morgan-Trimmer, Anne Marie Minihane, Penny Rapaport, Miguel Rio, Karen Ritchie, Zuzana Walker, Kate Walters, Rachael M Hunter

**Affiliations:** Centre for Psychiatry and Mental Health, Queen Mary University of London, London, UK; Centre for Psychiatry and Mental Health, Queen Mary University of London, London, UK; Division of Psychiatry, University College London, London, UK; Centre for Psychiatry and Mental Health, Queen Mary University of London, London, UK; Division of Psychology and Language Sciences, University College London, London, UK; Division of Psychiatry, University College London, London, UK; Department of Statistical Science, University College London, London, UK; Priment Clinical Trials Unit, Research Department of Primary Care and Population Health, University College London, London, UK; Department of Statistical Science, University College London, London, UK; Priment Clinical Trials Unit, Research Department of Primary Care and Population Health, University College London, London, UK; Facultad de Ciencias Biomédicas y de la Salud, Universidad Europea, Madrid, Spain; Centre for Healthy Brain Ageing, Discipline of Psychiatry and Mental Health, University of New South Wales, Sydney, New South Wales, Australia; Centre for Psychiatry and Mental Health, Queen Mary University of London, London, UK; Division of Psychiatry, University College London, London, UK; Faculty of Health and Life Sciences, University of Exeter, Exeter, UK; Department of Psychiatry and Behavioral Sciences, University California Davis, Davis, California, USA; National Institute for Health and Care Research (NIHR) Applied Research Collaboration the South West Peninsula (PenARC), University of Exeter, Exeter, Devon, UK; Division of Psychiatry, University College London, London, UK; Faculty of Medicine, University of Southampton, Southampton, UK; Norwich Medical School, University of East Anglia, Norwich, UK; Division of Psychiatry, University College London, London, UK; Department of Electronic and Electrical Engineering, University College London, London, UK; Inserm, Unit 1061, Neuropsychiatry: Epidemiological and Clinical Research, La Colombière Hospital, University of Montpellier, Montpellier, France; Division of Psychiatry, University College London, London, UK; Essex Partnership University NHS Foundation Trust, Essex, UK; Department of Statistical Science, University College London, London, UK; Priment Clinical Trials Unit, Research Department of Primary Care and Population Health, University College London, London, UK

**Keywords:** health economics, non-pharmacological, dementia, prevention, older people

## Abstract

**Background:**

Previous studies have modelled long-term cost-effectiveness of dementia prevention but seldom within trials. APPLE-Tree (Active Prevention in People at risk of dementia through Lifestyle, bEhaviour change and Technology to build REsiliEnce) is a personalised, multi-domain, low-intensity dementia prevention intervention for adults experiencing subjective cognitive decline or mild cognitive impairment. Intervention receipt, relative to control, was associated with an adjusted mean difference of 0.06 (95%CI −0.001 to −0.128) in the Neuropsychological Test Battery (NTB). We evaluated cost-effectiveness from health- and social-care perspectives over 2 years.

**Methods:**

We recruited 746 older adults from English health and community settings and randomly assigned them (1:1) to APPLE-Tree plus usual care (UC) or UC plus written dementia prevention information. Quality-adjusted life-years (QALYs) were calculated from the EQ-5D-5L. Over 24-months, intention-to-treat analyses compared QALYs (primary) and incremental cost per NTB unit (one z-score) change (secondary).

**Results:**

Between October 2020 and December 2022, 374 participants were randomised to intervention and 372 to control. Mean adjusted QALYs were 1.511 (intervention) and 1.520 (control), an adjusted difference of −0.010 (95%CI −0.04 to −0.022). Mean adjusted (SD) costs were £2966 (3629) and £2551 (4439), respectively. Cost per 1-point NTB change z-score was £6809. At a £30 000 threshold, the probability of cost-effective was 12% and 96% based on QALYs and cognition, respectively. Post hoc analyses indicated higher cost-effectiveness for non-White participants (incremental costs: £374; 95%CI −590.751 to −1338.515) and socioeconomically disadvantaged participants (non-homeowners), who had higher QALYs (0.106) and lower costs (−£1830), yielding a 98% probability of cost-effectiveness at a £20 000 threshold.

**Conclusion:**

APPLE-Tree has a high probability of being cost-effective for a 1-point NTB gain, a difference previously associated with a three-fold reduction in 5-year dementia risk. QALY-based cost-effectiveness was not observed, but post-hoc analyses suggested it may be more effective in groups with greater needs. Findings highlight the potential of targeted, lower-intensity interventions to reduce dementia risk. Longer-term follow-up is required to determine whether cost-effectiveness is realised over time.

## Key points

- Differences in cost-effectiveness based on QALYs were not statistically significant over 2 years.
- The APPLE-Tree intervention had a high likelihood of cost-effectiveness for attaining a significant change in cognition.
- It is plausible that APPLE-Tree will offer a cost-effective, scalable model for dementia prevention in high-risk populations.

## Introduction

Around 982 000 people in the UK, and 57 million people worldwide, have dementia [1, 2]. Total UK costs for dementia are projected to increase from £23.0 billion in 2015 to £80·1 billion in 2040 [3]. By 2030, worldwide costs are predicted to be an estimated US$1.7 trillion [1]. However, the Lancet Commission estimated that ~45% of dementia cases could potentially be prevented by addressing modifiable risk factors [4]. Delaying onset could also substantially reduce costs; e.g. a 5-year delay in Alzheimer’s disease onset has been estimated to reduce societal costs by ~40%, with savings of over $500 000 per affected individual [5]. There is, therefore, a pressing need to identify effective and implementable prevention strategies to realise these benefits.

We recently reported the findings from a co-designed secondary dementia prevention intervention (APPLE-Tree, Active Prevention in People at risk of dementia through Lifestyle, bEhaviour change and Technology to build REsiliEnce), that aimed to include groups often underrepresented in research [6–8]. This low-intensity, group-based intervention involves setting goals to target modifiable risk factors identified by the Lancet Commision, including physical activity, engaging with life (increasing pleasurable activities), connecting with others (increase social connections), reducing alcohol and smoking, promoting self-care of long-term physical conditions and improving sleep and mental wellbeing, as well as healthy diet and hydration. Many trials fail to target those at highest risk of dementia, including people from under-served groups [9]. In contrast, we targeted under-served individuals with objective (mild cognitive impairment, MCI) or subjective (subjective cognitive decline, SCD) memory concerns, who are at increased risk of progression to dementia [10]. These individuals also tend to use more healthcare resources relative to those with normal cognition [11], so we were also selecting a group in whom there was greater opportunity to reduce costs through prevention.

In our primary trial analysis, neuropsychological test battery (NTB) scores marginally favoured the APPLE-Tree intervention in individuals with subjective or objective cognitive decline over 24 months, relative to an informational control (intervention: *n* = 271, mean 0.21 [SD 0.75] versus control: *n* = 249, 0.33 [0.67]; adjusted mean difference 0.06 [−0.001 to 0.128], *P* = .055) [8]. These findings broadly align with those from other randomised controlled trials (RCTs), in which multimodal lifestyle interventions have been associated with small, positive changes on cognitive outcomes, relative to control conditions [12–14], although the APPLE-Tree trial did not reach statistical significance at 24 months (*P* = .055). The difference in total NTB score we observed, which approached statistical significance, is of similar magnitude to that reported in the FINGER [13] and US POINTER [14] trials, despite those studies involving more intensive interventions delivered over 2 years, compared with 1 year in APPLE-Tree. Cognitive effects of this magnitude, as estimated from FINGER trial data, have been equated to a relative 20-year dementia risk reduction of around 6% [15]. In a Markov model, simulating an initial cohort of 100 000 persons at risk for dementia over 30 years, the authors estimated that 1623 dementia cases could be avoided [16]. In a 20 year simulation using FINGER findings, the cost was 21 974 SEK lower with the intervention, with 0.0511 quality-adjusted life years (QALYs) gained [17]. Other economic simulations also suggest that dementia prevention is cost-effective, longer-term [18].

To our knowledge, only two previous RCTs have reported cost-effectiveness of preventive multidomain interventions using actual, rather than simulated costs. Over 3 years, the Multidomain Alzheimer Preventive Trial (MAPT) found a non-significant trend towards effectiveness of a multidomain intervention compared with placebo in people living with cognitive or physical frailty. The intervention Incremental Cost Effectiveness Ratio (ICER; the additional cost per unit of effect gained) was €21 543 per improved cognitive z-score point (on a composite measure combining four cognitive tests) compared with placebo [19]. The Maintain Your Brain trial examined the effect of a multi-domain internet-based dementia prevention program for people aged 55–77 from the general population and reported an ICER of $AUS 2568 per cognitive z-score point over 3 years [20].

In people at risk of dementia, a one-point change in cognitive composite z-score has been estimated as equivalent to a three-fold reduced risk of developing dementia over 5 years in people at risk of dementia [21]. Other trials have reported actual interventions costs without conducting full cost-effectiveness analyses [12, 22], while some have estimated cost-effectiveness using simulated data that extrapolate short-term trial effects of multidomain interventions [16]. However, whilst these approaches have utility (e.g. in estimating longer-term outcomes when it is often difficult to follow participants for the duration required to observe effects on cost-effectiveness in prevention trials), they also have limitations as they assume that risk reductions from trial populations can be extrapolated to real-world, at-risk populations, and that intervention effects will not wane after delivery ends [23].

This is only the third study to report within-trial, clinical effectiveness of a multidomain, dementia prevention intervention. We report findings of our secondary APPLE-Tree trial objectives, to calculate the mean incremental cost (i) per QALY and (ii) per unit change in NTB cognitive z-score, of the APPLE-Tree intervention compared with the control condition [usual care (UC) plus written information about dementia prevention] in individuals at high risk of dementia (with subjective or objective memory concerns) from the English NHS and Personal Social Services (PSS) perspective. We also report exploratory analyses examining whether cost-effectiveness varies by socioeconomic status and ethnicity.

## Methods

### Study design

This is a cost-utility and cost-effectiveness analysis conducted within a two-armed, parallel-group, single-masked, superiority randomised controlled trial across 11 sites in England. The study was preregistered with the ISRCTN Registry on 27 November 2019 (ISRCTN17325135) and the trial protocol was published [24]. Ethical approval was granted by London (Camden and Kings Cross) Research Ethics Committee (Reference: 20/LO/0034) and the UK Health Research Authority (HRA) approved the study in February 2020.

### Participants

We recruited adults aged 60 and older with SCD or MCI through multiple channels: NHS primary care practices and memory clinics within 2 h of London or Essex (with sites purposively selected for ethnic and sociodemographic diversity), the recruitment database Join Dementia Research, non-governmental organisations for older people, X (formerly Twitter), the APPLE-Tree website, and local and national newspapers. Sex (with options: male, female or other [to specify]) and ethnicity (using Office of National Statistics categories) and home ownership were all self-reported. Full eligibility criteria are described elsewhere [8].

### Randomisation and masking

Participants were randomly allocated (1:1) to the intervention or control group. Allocations were obtained through a remote web-based system (www.sealedenvelope.com) provided by PRIMENT Clinical Trials Unit (CTU). Randomisation was stratified by recruitment site and used blocks of varying length. Outcome assessors were blinded to group allocation. However, due to the nature of the intervention and clustering within the intervention arm, participants, informants, facilitators, statisticians and health economists could not be blinded.

### Procedures

Assessments were conducted via video call; from April 2021, when COVID-19 restrictions were lifted in England, in-person assessments were also offered. Data were collected at baseline, 12 months and 24 months.

APPLE-Tree was delivered in pairs by facilitators without clinical qualifications; by a university-based researcher and a facilitator from an non-governmental organisation (NGO) working with older people at community sites; or by two assistant psychologists at NHS sites. Facilitators delivered 10 manualised 1-h group video-call sessions held fortnightly over six months with groups of four to nine participants, and a 40 min group ‘tea break’ (i.e. unstructured, informal social sessions) in the weeks between intervention sessions. Participants also received a phone call (up to 30 min) after each main session from a facilitator, to discuss and set new or revise existing goals. From months 6 to 12, participants continued with monthly online ‘tea breaks’. A random 10% of intervention sessions were selected for audio recording (subject to consent of all participants) and used to assess fidelity to the intervention manual [25].

All participants in the control arm received a booklet about dementia prevention produced by the Alzheimer’s Society; participants in both groups received routine care (no specific therapeutic interventions are offered in England, with care typically consisting of signposting, assessment, monitoring, general health and lifestyle advice, and referral to memory services if indicated).

### Cost of the intervention

The cost of APPLE-Tree included the time for the facilitators to prepare for and deliver the intervention, plus the cost of training and supervision of the facilitators by a senior researcher. Hourly rates for facilitators and a supervising clinical psychologist and nutritionist were based on university salary scales for the respective grades and included oncosts and overheads. Total training and supervision costs were divided by the number of participants in the treatment group to produce a unit cost per participant. Treatment as usual comprised dementia prevention information plus UC, based on self-reported primary and secondary healthcare service use.

### Resource use and costs

Resource use in both groups was collected from self-reported questionnaires at baseline, after 12 months, and after 24 months using a modified version of the Client Service Receipt Inventory (CSRI) [26]. The CSRI asked about secondary care contacts (planned and unplanned), emergency services, primary care, community, medication, and accommodation, including out-of-pocket costs for private care home accommodation in the last 6-months. Resource use was costed using the Personal Social Service Resource Unit (PSSRU) Unit Costs [27], NHS Reference Costs [28] and the published literature (see Appendix 1 in the Supplementary Material). All costs are reported in British Pounds (GBP) for the year 2023/2024.

### Outcomes

The primary clinical outcome, previously reported, was cognition at 24 months assessed via the NTB, summarised as a z-score where higher scores reflect better cognitive performance. The NTB is highly sensitive to change, has excellent internal consistency and test–retest reliability [29] and has been validated for video-call delivery [30].

Participants were asked to complete the EQ-5D 5 level (EQ-5D-5L) [31] at baseline, 12-months and 24-months to allow for the calculation of QALYs. For the primary health economic analysis EQ-5D-5L scores were converted to utility scores using the algorithm to map EQ-5D-3L to EQ-5D-5L [32]. A secondary analysis was conducted using the value set for England (VSE) to calculate utility scores [33].

### Statistical analysis

The principal economic analysis was estimation of the change in incremental cost per QALY of the APPLE-Tree intervention compared with the control at 24 months, adjusting for baseline utilities, costs and site. All analyses used the intention-to-treat principle. Analyses were conducted using Python version 3.12, with MissForest used to impute missing data based on the assumption that data were missing at random [34]. The full health economic analysis plan is presented in Appendix 1 in the Supplementary Material.

QALYs were calculated from participant responses to the EQ-5D-5L using the area under the curve at each follow-up [35]. For participants who died between follow-ups, a QALY value of 0 was assigned from the date of death onwards. Mean utility values for both groups were calculated at baseline, 12 months, and 24 months. We estimated the incremental QALY (comparing APPLE-Tree and control arms) using a linear regression model to adjust for baseline utility score and site as fixed effects. We found no significant effect for clustering by therapy group, so this was not included in the analysis.

We estimated the mean incremental cost per QALY gained between the APPLE-Tree intervention and control arm at 24 months using 5000 iterations of two-stage bootstrap and bias corrected and accelerated (BCa) standard error to allow for the non-parametric assumption in the bootstrapping procedure. We calculated the adjusted mean and adjusted incremental (differences) in costs and QALYs between the intervention and control arm and 95% confidence intervals, again using BCa approach (adjusting for baseline utilities, baseline costs and site as fixed effects). A 3.5% annual discount rate for costs and outcomes between 12 and 24 months was applied in accordance with NICE guidance.

The bootstrapped increments for costs and QALYs were used to calculate the probability that the APPLE-Tree intervention was cost-effective compared with the control arm for a range of cost-effectiveness thresholds for one QALY gained at 24 months and to generate the cost-effectiveness plane (CEP). We also assessed the incremental cost per unit change in NTB z-score by calculating a CEP and cost-effectiveness acceptability curve (CEAC), using the same methods described above. Although this analysis was not specified in the original HEAP unless a statistically significant effect on NTB was observed, it was conducted as an exploratory secondary analysis because the primary analysis approached significance (*P* = .055).

In addition to the primary and secondary cost-effectiveness analyses, we conducted exploratory post-hoc analyses to examine whether results varied by participant characteristics, in order to inform implementation of APPLE-Tree as a potential intervention to reduce stark inequalities in dementia prevention. Specifically, we investigated whether cost-effectiveness differed according to socioeconomic status, defined by self-reported home ownership (homeowner versus non-homeowner), and ethnicity, categorised as White versus non-White.

Our *a priori* sample size calculation indicated 704 participants (352 per arm) was sufficient to detect, with 90% power and 5% significance, a difference of 0.15 on NTB score (effect size = 0.25, SD 0.6) between intervention and control groups at 24-months. This calculation allowed for baseline adjustment (assumed correlation coefficient 0.6), intervention group clustering (intracluster correlation coefficient [ICC] 0.03, design effect 1.93); and 10% loss to follow-up.

## Results

Between 5 October 2020 and 31 December 2022, 748 participants were recruited. One participant was mistakenly randomised twice and excluded after Trial Steering Committee review, resulting in 374 participants randomised to the intervention and 372 to the control group. The CONSORT diagram is shown Appendix 2 of the Supplementary Material. The primary outcome analysis included 635/746 (85%) participants who had at least one NTB measurement post-randomisation. Table S1 shows the participant baseline characteristics (see Appendix 3 in the Supplementary Material).

### Intervention costs

Twenty university or NHS employed facilitators (£25.51 per hour) and 11 voluntary, social and community organisation workers (£18 per hour) each spent an average of 15 h in training (including session practice and preparation time) and 15 h in supervision groups (total cost £28 334). The intervention was delivered to 55 therapy groups, who each received 10 main sessions (1 h) plus 10 tea breaks (40 min) and 6 further monthly tea breaks (40 min), delivered by pairs of university and voluntary, social and community organisation workers, or pairs of NHS workers (total cost £51 941). In addition, intervention participants received on average 7.4 goal calls, lasting on average half an hour, from one facilitator (cost £31 619). A clinical psychologist worked 2 h per week (for 40 weeks per year, from November 2020 to July 2022 [21 months]) to provide training and supervision at an hourly rate of £58.50 (total cost £9360). A nutritionist was paid £75 per month from February 2021 to April 2023 (27 months) to deliver supervision groups. All participants received a step counter (£20), one-off food delivery (£18.45) and a printed manual and goal booklet (£9.32 plus postage). The APPLE-Tree intervention mean cost per participant was £359.

### Costs of control condition

The cost of the control condition comprised the printing and postage of dementia prevention information (total cost per participant £2).

### Comparing intervention and control costs

Costs at baseline, 12 months, and 24 months by allocation group are reported in Table 1 and Appendices 3 and 4 in the Supplementary Material. Data to calculate total health and social care costs were available for 733 participants at baseline, 580 (79%) participants at 12 months and 508 (69%) participants at 24 months.

**Table 1:** Summary of health care costs in intervention and control arms.

Variable	Time point	Intervention (mean, SD)	*N*	Control (mean, SD)	*N*	Difference (95% confidence interval)^a^
Emergency care costs	0	£49 (163)	375	£72 (186)	373	−22 (−93 to 12)
	12	£42 (160)	290	£50 (165)	290	−9 (−44 to −8)
	24	£40 (143)	267	£64 (203)	241	−24 (−31 to −11)
Outpatients	0	£656 (1616)	375	£958 (2678)	373	−303 (−1007 to 646)
	12	£610 (1599)	290	£399 (1047)	290	211 (−1258 to 203)
	24	£503 (1253)	267	£795 (2563)	241	−294 (−1251 to 63)
Unplanned admissions	0	£80 (547)	375	£194 (1504)	373	−113 (−774 to −139)
	12	£95 (920)	290	£58 (475)	290	38 (−366 to −245)
	24	£49 (398)	267	£81 (574)	241	−46 (−262 to −64)
Planned admissions	0	£182 (1144)	375	£100 (906)	373	85 (−234 to −361)
	12	£116 (842)	290	£133 (1010)	290	−16 (−514 to −278)
	24	£116 (842)	267	£167 (1194)	241	−53 (−225 to −292)
GP	0	£35 (60)	375	£31 (43)	373	4 (−26 to 8)
	12	£29 (47)	290	£36 (83)	290	−6 (−79 to 49)
	24	£32 (60)	267	£35 (53)	241	−3 (−43 to 28)
Primary-care Nurse	0	£8 (15)	375	£8 (14)	373	1 (−7 to −3)
	12	£8 (23)	290	£8 (18)	290	0 (−13 to −1)
	24	£8 (14)	267	£11 (22)	241	−3 (−6 to −2)
Community Nurse	0	£3 (22)	375	£3 (32)	373	−1 (−17 to −6)
	12	£0 (2)	290	£4 (67)	290	−4 (−32 to −7)
	24	£0 (3)	267	£4 (74)	241	−4 (−29 to −14)
HCA	0	£1 (3)	375	£0 (3)	373	0 (−1 to −1)
	12	£0 (2)	290	£0 (3)	290	0 (−1 to −1)
	24	£0 (3)	267	£1 (4)	241	0 (−2 to −1)
CMHT	0	£14 (105)	375	£30 (242)	373	−17 (−14 to −18)
	12	£5 (45)	290	£23 (251)	290	−18 (−188 to 44)
	24	£22 (284)	267	£47 (506)	241	−27 (−215 to −106)
Other community care	0	£63 (268)	375	£40 (135)	373	22 (−89 to −22)
	12	£49 (167)	290	£34 (93)	290	15 (−90 to −30)
	24	£52 (198)	267	£46 (141)	241	6 (−102 to −18)
Therapy	0	£45 (397)	375	£24 (373)	373	22 (−185 to −114)
	12	£5 (29)	290	£3 (31)	290	2 (−40 to 16)
	24	£10 (113)	267	£8 (78)	241	2 (−59 to −24)
Optician	0	£41 (83)	375	£45 (70)	373	−5 (−14 to 11)
	12	£39 (76)	290	£37 (66)	290	2 (−41 to 21)
	24	£40 (72)	267	£43 (75)	241	−3 (−32 to 1)
Social Worker	0	£1 (7)	375	£1 (10)	373	0 (5 to −3)
	12	£2 (19)	290	£1 (7)	290	1 (−8 to −5)
	24	£0 (5)	267	£0 (3)	241	0 (1 to −1)
Pharmacist	0	£34 (248)	375	£19 (75)	373	16 (−110 to −42)
	12	£27 (182)	290	£32 (190)	290	−5 (−188 to 32)
	24	£30 (115)	267	£30 (95)	241	0 (−88 to 11)
Home Care	0	£65 (768)	375	£36 (373)	373	31 (−334 to −198)
	12	£15 (259)	290	£14 (237)	290	1 (−137 to −76)
	24	£24 (321)	267	£19 (143)	241	4 (−102 to −77)
Medication	0	£38 (34)	375	£36 (30)	373	3 (−21 to 13)
	12	£42 (38)	290	£37 (32)	290	5 (−19 to 9)
	24	£41 (36)	267	£38 (32)	241	4 (−24 to 10)
Total imputed cost	0	£1309 (2536)	375	£1591 (3797)	373	−279 (−748 to 190)
	12	£1072 (2411)	375	£857 (1868)	373	215 (−96 to 526)
	24	£938 (1944)	375	£1374 (3566)	373	−443 (−859 to −27)
	12 + 24 + RH	£2563 (3629)	375	£2629 (4439)	373	52 (−507 to 610)
	12 + 24+ RH + intervention	£2922 (3629)	375	£2631 (4439)	373	409 (−150 to 967)
	12 + 24+ RH + intervention (adjusted means)	£3016 (3629)	375	£2607 (4439)	373	409 (−150 to 967)
	12 + 24+ RH + intervention discounted	£2874 (3629)	375	£2574 (4439)	373	416 (−130 to 962)
	12 + 24+ RH + intervention discounted (adjusted means)	£2966 (3629)	375	£2551 (4439)	373	416 (−130 to 962)

GP, general practitioner; HCA, health care assistant; CMHT, community mental health team; RH, local authority residential home; SD, standard deviation

^a^Adjusting for baseline and site

Table 1 also shows imputed, adjusted total mean costs. After imputation, the total mean adjusted health and social care cost per participant at 24 months, including the cost of training and delivering APPLE-Tree, was £3016 (£2966 discounted SD 3629) in the intervention group compared with £2607 (discounted £2551 SD 4439) in the control group, a difference of £409 (95% Confidence Interval [CI] −150 to 967) (£416 [95% CI −130 to 962 discounted]); this difference is not statistically significant.

### Quality-adjusted life-years and cognition

The mean utility scores regarding participant quality of life (EQ-5D-5L) are reported in Table 2, both before and after imputation. There were complete data to calculate the EQ-5D-5L for 730 (97.9%) participants at baseline, 577 (77.3%) participants at 12 months, and 512 (68.6%) participants at 24 months. A zero was imputed for the 13 participants who died. Four hundred and sixty-four (62.2%) participants had data available to calculate QALYs across the 24 months. The mean adjusted and imputed QALY at 24 months was 1.538 (discounted 1.511 SD 0.329) for participants randomly assigned to the APPLE-Tree intervention and 1.547 (1.520 discounted SD 0.338 for those assigned to the control, with a mean difference in QALYs of −0.010 (95% CI −0.042 to 0.022), adjusting for baseline utilities and site.

**Table 2:** Summary of EQ-5D in intervention and control arms, complete and imputed data.

Variable	Complete data		Imputed data	
	Intervention (Apple-Tree)	Control	Intervention (Apple-Tree)	Control
EQ-5D baseline n	363	367	365	367
EQ-5D baseline mean(SD)	0.774 (0.199)	0.765 (0.212)	0.773 (0.199)	0.765 (0.212)
EQ-5D 12-months n	288	289	365	367
EQ-5D 12-months mean(SD)	0.763 (0.222)	0.77 (0.219)	0.767 (0.199)	0.772 (0.197)
EQ-5D 24-months n	241	271	365	367
EQ-5D 24-months mean(SD)	0.782(0.218)	0.772 (0.237)	0.783 (0.18)	0.776 (0.205)
EQ-5D total QALYs n	221	243	365	367
EQ-5D total* QALYs mean(SD)	1.559(0.377)^a^	1.548(0.384)^a^	1.545 (0.335)	1.543 (0.345)
EQ-5D total QALYs adjusted mean(SD)			1.538 (0.335)	1.547 (0.345)
EQ-5D total QALYs discounted mean(SD)			1.517 (0.329)^b^	1.516 (0.338)^b^
EQ-5D total QALYs discounted adjusted mean(SD)			1.511 (0.329)	1.520 (0.338)

^a^mean difference (95% confidence interval): 0.0091(−0.035 to 0.053) (*P* = .713) (complete data); ^b^ −0.01(−0.041 to 0.022) (*P* = .554) (imputed data)

**Figure 1:**
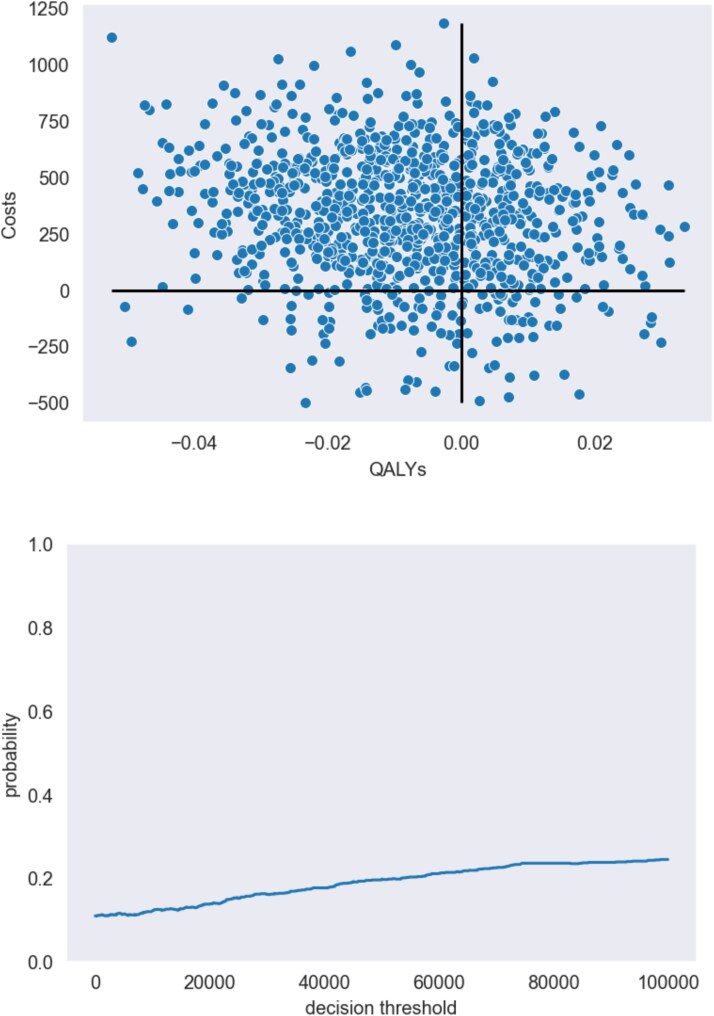
Estimates from two part bootstrap using MissForrest imputation data and adjusting for baseline, site, discounted, EQ-5D-5L and mapping algorithm; QALY, quality-adjusted life-year.

**Figure 2:**
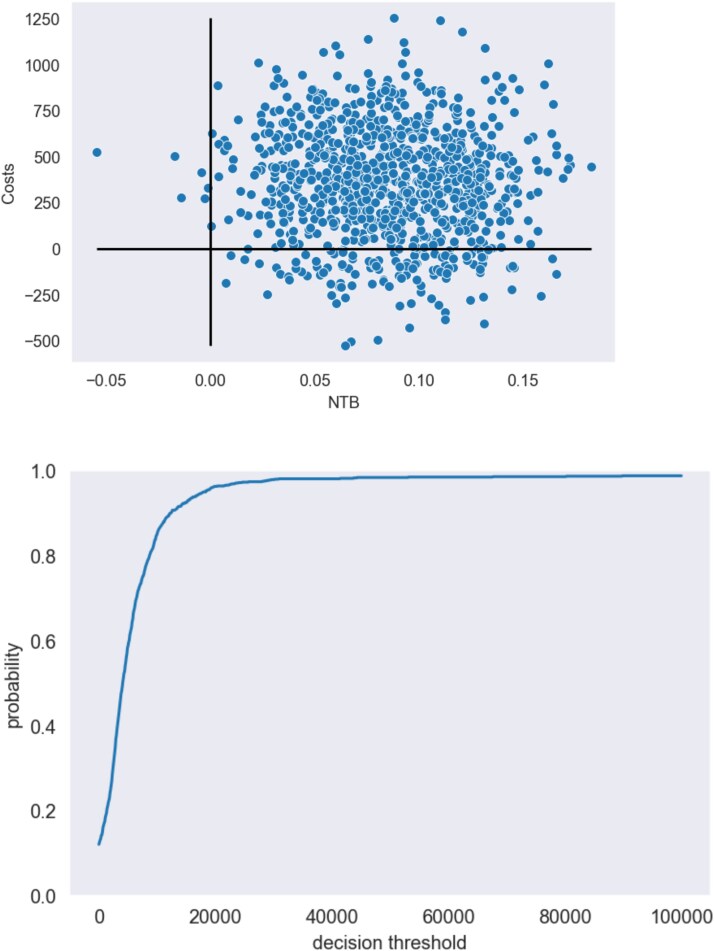
Two part bootstrap using multiple imputation data and adjusting for baseline, site and facilitator clustering; NTB, neuropsychological test battery score.

APPLE-Tree is dominated by control as it results in fewer QALYs for a higher cost, although the differences are not statistically significant. The mean incremental cost per 1-point change in NTB z-score was £6809. At a £30 000 decision threshold, the probabilities that APPLE-Tree is cost-effective are 12% based on QALYs (Fig. 1) and 96% based on cognition (Fig. 2).

### Exploratory analyses: socioeconomic status and ethnicity

APPLE-Tree was highly cost-effective for participants who did not own their homes, with an incremental QALY gain of 0.106 (95% CI −0.002 to 0.214) and lower costs of £1830 (95% CI −3701 to 41), resulting in it being dominant and a 98% probability of being cost-effective at £20 000 and £30 000 thresholds. For non-White participants, APPLE-Tree was associated with a small incremental QALY gain of 0.004 (95% CI −0.096 to 0.105) and incremental costs of £374 (95% CI −591 to 1339), corresponding to an ICER of £83 220 per QALY. At decision thresholds of £20 000 and £30 000, the probability of cost-effectiveness in this subgroup was 43% and 48%, respectively, indicating a slightly higher likelihood of cost-effectiveness relative to White participants.

## Discussion

The APPLE-Tree intervention has a low (12%) likelihood of being cost-effective in terms of QALYs but a high (96%) likelihood of cost-effectiveness in terms of improving cognition, compared with informational control, over 2 years. Our analysis of cognition was based on improvement of one z-score point on a composite measure. This difference is important because people in a similar population who experienced a one-point decrease in z-score on this measure were ~3.5 times more likely to be diagnosed with dementia [21]. Costs were similar between intervention and control groups, but the future costs of dementia are high, with an annual cost per person of £28 700 for mild dementia and £80 500 for severe [36]. Exploratory analyses indicated that the intervention was particularly cost-effective for socioeconomically disadvantaged participants and showed higher, but more limited, probability of cost-effectiveness for non-White participants. These findings indicate that targeted lifestyle interventions may be particularly valuable in higher-need groups. We are following participants for ~5 years, and if the effects of the APPLE-Tree intervention are sustained over time resulting in the prevention or delay of dementia, the probability of cost-effectiveness may well increase [37].

National governments are shifting focus of health policies from sickness to prevention: this is a pillar of the UK government’s Health Mission [38]. If health systems are to incorporate lifestyle-change interventions that are sustainable and broadly effective, these need to be deliverable at scale and work across conditions. Based on this, it is noteworthy that, in addition to the changes we have reported here, APPLE-Tree intervention-arm participants also significantly improved on measures of healthy diet [8]. A 2-point increase in adherence score to the Mediterranean diet (measured using the Mediterranean diet score) predicted reductions of 8% in mortality, 10% cardiovascular disease and 4% risk of cancer in a recent meta-analysis using a 9-point scale [39]. There is good reason to suppose that the benefits of APPLE-Tree, for both quality of life and general health, will continue to accrue over time: models developed for the National Institute for Health and Care Excellence (NICE) guidelines for diabetes prevention assume cardiometabolic health gains persist for 5 years [40].

This is only the third within-trial cost-effectiveness evaluation of a non-pharmacological dementia prevention intervention; both previous studies reported the ICER for a 1-point cognitive z-score point change. Our finding that the intervention has a high likelihood of being cost-effective in delivering a significant change in cognition using this outcome is promising, though several limitations should be considered when interpreting these results. As in all psychological treatment trials, participants could not be blinded to allocation. The 2-year trial follow-up allowed assessment of cognition but not dementia incidence, and changes in quality of life may also take longer to accrue. Dementia prevention trials may not always provide sufficient time for true effects to emerge. Previous studies have extended trial findings to estimate the impact of dementia prevention interventions in real-world populations, but these estimates rely on assumptions that have been disputed—such as that intervention effects will persist for years beyond intervention delivery, and that real-world populations engage with interventions in similar ways to trial populations [23]. The economic evaluation was conducted from a health and PSS perspective, in line with NICE guidance, and did not include informal caregiver costs. As a result, the broader societal impact of the APPLE-Tree intervention may be underestimated, given the importance of unpaid care in dementia. The trial population were, as noted in our previous qualitative work in a liminal position between independence and requiring care from family members. Indeed, many were themselves providing care to others [25]. Future dementia prevention trials should consider how to capture the broader societal and informal care costs to enhance the relevance of findings for prevention-focused policy decisions. Attrition over follow-up may have introduced potential bias, as participants with missing data were generally older, less educated, had lower baseline NTB scores, and were less likely to be married or living with a partner compared with those with complete 24-month data. In APPLE-Tree, differences in cost-effectiveness based on QALYs were not statistically significant over 2 years; we are collecting longer-term data in a trial extension and will use decision modelling to assess longer-term cost-effectiveness.

APPLE-Tree was designed to be inclusive. Compared with census data for adults aged 65 years and older, we included more participants from non-White ethnic groups (11.6% versus 6.4%). However, the study population was highly educated and more likely to own their home relative to the general population. Against this backdrop, exploratory analyses are informative: the intervention was particularly cost-effective for socioeconomically disadvantaged participants (non-homeowners) and had a higher, but more modest, probability of cost-effectiveness among non-White participants. These patterns suggest that the benefits of APPLE-Tree may be underestimated in this trial and could be greater when implemented in more socioeconomically diverse, real-world populations, where dementia risk is often higher and research participation typically lower [41]. We estimated costs every six months over 2 years, so actual costs in both arms would also be higher.

This is a critical and hopeful time for dementia prevention. Although APPLE-Tree did not demonstrate statistically significant differences in cognition or QALYs at 24 months in the primary analyses, the direction and magnitude of effects, alongside the high probability of cost-effectiveness for cognitive outcomes, suggest potential value that may emerge over longer follow-up. If and when disease-modifying treatments for dementia become widely available, they will have the potential to delay disease progression, including conversion from MCI to dementia, in a proportion of those with Alzheimer’s disease who are eligible and can tolerate new drugs. The advent of such treatments is also likely to drive earlier dementia diagnosis. In this context, strengthening the evidence base for non-pharmacological therapies to complement those new treatments, and to provide alternatives where they are not appropriate or beneficial, is of heightened importance. Alongside longer-term follow-up of our participants, we will now explore how to support implementation of our findings into practice.

## Supplementary Material

aa-25-3491-File002_afag176

## Data Availability

Data collected for the study, including the statistical analysis plan, de-identified participant data and a data dictionary defining each field in the set, will be made available to others on receipt by Priment Clinical Trials Unit ([CTU] priment@ucl.ac.uk) of a reasonable request at any date after publication of this Article. All requests will be reviewed by Priment CTU in line with Priment CTU guidance on sharing data and anonymising data. This process is to ensure that the request is reasonable and that the dataset is suitably anonymised. The study protocol is available on an open-access basis. Intervention materials are available without cost, subject to a CC BY-NC-ND licence held by CC.
