# Supplementary material for: Cost utility and cost-effectiveness of the APPLE-Tree programme: Active Prevention in People at risk of dementia through Lifestyle, bEhaviour change and Technology to build REsiliEnce: economic evaluation embedded within a randomised controlled trial
Source: Age Ageing. 2026 Jun 22;55(6):afag176. doi: 10.1093/ageing/afag176 (PMC13284706; doi:10.1093/ageing/afag176)
Supplement: aa-25-3491-File002_afag176 [file aa-25-3491-file002_afag176.docx]

**Supplementary Material for Cost utility and cost-effectiveness of the APPLE-Tree programme: Active Prevention in People at risk of dementia through Lifestyle, bEhaviour change and Technology to build REsiliEnce: economic evaluation embedded within a randomised controlled trial**

**Appendix 1:** The APPLE Tree trial: Health Economic Analysis Plan (HEAP)

**Appendix 2:** Supplementary material figure 1 - Trial CONSORT diagram

**Appendix 3:** Supplementary material table 1 - Baseline participant characteristics by arm

**Appendix 4:** Supplementary material table 2 - Descriptive statistics for community, primary and secondary health and social care resource use

**Appendix 5:** Supplementary material table 3 - Descriptive statistics for inpatient resource use

**
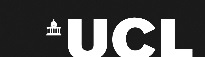

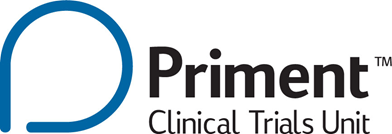
**

**The APPLE Tree trial: Health Economic Analysis Plan (HEAP)**

Full title of trial: **Active Prevention in People at risk of dementia through Lifestyle, bEhaviour change and Technology to build REsiliEnce: Pilot of the intervention and Randomised Controlled Trial (Phase 2)**

**Version 1.3**

Date: 3rd April 2025

Prepared by: Lucie Teoh Email: l.teoh@ucl.ac.uk

Based on SAP v1.0 (October 2024)

**Version history:** Version 1.2 has been reviewed by Claudia Cooper, Rachael Hunter and Harriet Demnitz-King and the TSC, including Julie Barber

| **Approved by:** | |  | | **Signature** | |  | | **Date** | |
| --- | --- | --- | --- | --- | --- | --- | --- | --- | --- |
| **Prof Claudia Cooper**  Chief Investigator | |  | |  | |  | |  | |
| Prof. Rachael Hunter  Co-app Health Economist | |  | |  | |  | |  |  |
| Dr Lucie Teoh  Trial Health Economist | |  | |  | |  | |  |  |
| Dr Julie Barber  Co-app Statistician | |  | |  | |  | |  |  |
|  | |  | |  | |  | |  |  |

Table of Contents

[1. Aim 4](#_Toc194579127)

[1.1 Purpose and scope of plan 4](#_Toc194579128)

[1.2 Health Economic Objective(s) 4](#_Toc194579129)

[2. Analysis Plan 4](#_Toc194579130)

[2.1 Perspective 4](#_Toc194579131)

[3. Resource use & costs 5](#_Toc194579132)

[3.1 Health service and social service use and costing 5](#_Toc194579133)

[4. Outcomes 6](#_Toc194579134)

[4.1 EQ-5D-5L 6](#_Toc194579135)

[4.2 Discounting 7](#_Toc194579136)

[5. PRIMARY ANALYSIS 7](#_Toc194579137)

[5.1 Incremental cost-effectiveness ratio (ICER) 7](#_Toc194579138)

[5.2 Cost effectiveness acceptability curve (CEAC) and cost-effectiveness plane (CEP) 8](#_Toc194579139)

[5.3 Missing data 8](#_Toc194579140)

[5.4 Sensitivity analyses 8](#_Toc194579141)

[6. SECONDARY ANALYSIS 9](#_Toc194579142)

[REFERENCES 10](#_Toc194579143)

Abbreviations

APPLE-TREE trial: Active Prevention in People at risk of dementia through Lifestyle, bEhaviour change and Technology to build REsiliEnce

CSRI: Client Service Receipt Inventory

CEA: Cost effectiveness analysis

CEAC: Cost effectiveness Acceptability Curve

CEP: Cost effectiveness Plane

ICER: Incremental cost-effectiveness ratio

MCI: Mild Cognitive Impairment

NICE: National Institute of Clinical Excellence

NTB: Neuropsychological Test Battery

QALY: Quality Adjusted Life Year

SAP: Statistical Analysis Plan

SCD: Subjective Cognitive Decline

TAU: Treatment as Usual

## Aim

This Health Economics Analysis Plan (HEAP) outlines the rationale, methods and planned analysis to achieve one of the secondary objectives of the APPLE-TREE trial (as laid out in the trial protocol v4 and the SAP), which is to evaluate the cost-effectiveness of the APPLE-TREE intervention in individuals with MCI or SCD at 24 months follow up.

### 1.1 Purpose and scope of plan

The primary aim of this economic evaluation is to estimate the mean incremental cost per quality adjusted life year (QALY) of the APPLE-Tree intervention^1^ compared with usual care (plus written information about dementia prevention) in individuals at high risk of dementia from the English NHS and Personal Social Services (PSS) perspective.

1.2 Health Economic Objective(s)

To evaluate the cost-effectiveness of the APPLE-Tree intervention in individuals with MCI or SCD at 24-month follow-up.

The principal analysis will calculate the incremental cost per quality adjusted life year (QALY) gained by the APPLE-Tree intervention compared to the control, adjusting for baseline differences in utilities and costs using regression analysis.

All health economic analyses will follow the assumptions outlined in the statistical analysis plan in terms of methods to assess and deal with missing data and loss to follow up. As such the economic evaluation will follow an intention-to-treat approach as per the APPLE-Tree protocol.

## Analysis Plan

### 2.1 Perspective

All economic analyses will be carried out from an English NHS and Personal Services perspective, using patient level trial data.

## Resource use & costs

### 3.1 Health service and social service use and costing

Primary and secondary healthcare service use (including hospital and community) and social care use will be collected using a modified version of the client service receipt inventory (CSRI) tailored for this population at 6 months pre-baseline, baseline, 12 months and 24 months. Information on medication use, accommodation use (e.g. nursing home, respite care) and out of pocket costs are also included in the modified CSRI.

The cost of hospital and community based secondary health care use for the APPLE-Tree versus control groups will be calculated from patient (or carer assisted) completed CSRI administered at baseline, 12 months and 24 months. As mentioned in the protocol, the baseline CSRI collects healthcare use data 6 months prior to each follow up time point.

Published national sources of unit costs will be used to calculate costs of health and social care service use including NHS reference costs, the British National Formulary (BNF)^2^ and the Personal Social Services Research Unit (PSSRU)^3^. For accommodation costs that are not readily available through the PSSRU, unit costs will be obtained from other published sources.

Descriptive statistics for patients using healthcare resource use will be presented for each type of healthcare use (e.g.secondary care, community care etc). Proportions of missing CSRI responses will be presented, following principles set out in the SAP.

Incremental differences in costs will be calculated using mixed effects regression, adjusting for baseline costs and site as fixed effects and allowing for clustering on therapy group^4^ (included as a random effect for those in the intervention arm) and with participants in the usual care group each considered as an individual cluster of size 1 (this is following the same methods used in the SAP. A fixed effects model will be used if the model fails to converge or if there is no evidence for clustering by therapy group). The incremental difference in costs between the intervention and usual care arms and 95% confidence intervals will be calculated based on bootstrapped results.

### 3.2 Cost of the Apple-Tree intervention and usual care

The mean cost of delivering the APPLE-Tree programme per person will be calculated by multiplying the unit cost of the trained facilitators that delivered the intervention (in person or via video call) by the duration and number of sessions that the facilitators deliver as well as the time spent on any catch-up calls. The cost will also include the cost of supervision by the clinical psychologist, nutritionist and study team. Information on the staff grade, duration of sessions, mode of delivery of session (home visit vs video call), travel time, as well as any other details including hours taken to train the facilitators and any supervision will be obtained from the trial team where not specified in the study protocol. We will account for intervention delivery and supervision group activities when calculating unit cost. We will also include the cost of any consumables (such as pedometers, food delivery, and manuals/books) given to participants. The cost of creating and maintaining the study website and study application were considered to be sunk costs so will not be included in the cost of the intervention. The total cost of the intervention will then be divided by the number of participants randomised to the intervention arm to provide a conservative estimate of the cost per participant to deliver APPLE-TREE (with the cost assuming to be lower with a higher number of participants). We will conduct a sensitivity analysis using a case load costing model (the cost to deliver the intervention to a specified number of participants based on the required inputs) rather than a cost per patient costing model.

The cost of the control (consisting of usual care plus written information about dementia prevention, including behavioural change targets with signposting information) will be calculated by the printing cost per page multiplied by the number of pages of written information given to patients as well as the cost of second class postage. Usual care will also include services collected as part of the CSRI such as GP and other specialist appointments.

## Outcomes

A full description of all study outcomes is provided in the trial protocol and statistical analysis plan. The following outcomes will be used for the economic evaluation carried out alongside the randomised control trial:

### 4.1 EQ-5D-5L

EQ-5D-5L is the primary economic outcome measure for this trial. The EQ-5D-5L^5^ is a generic measure of health-related quality of life, an easy to complete and administer 5 item questionnaire consisting of 5 levels, scored 1 to 5.

QALYs will be calculated based on the EQ-5D-5L responses completed by the patient in questionnaire format at baseline, at 12 months and 24 months. QALYs will be calculated using the area under the curve applied at each point of follow up.

We will map EQ-5D-5L utility scores onto EQ-5D-5L UK population value set using the algorithm reported by Hernandez Alava and colleagues^6^. This process is conditional on age and sex so utility scores will only be able to be calculated for participants with complete information on EQ-5D-5L, age and sex. For a secondary analysis we will calculate utility scores using the value set for England published by Devlin^7^ and colleagues and report them separately.

We will report mean utility values and standard deviation and mean unadjusted QALYs and standard deviations for the APPLE-Tree intervention group and control group at baseline, 12 months and 24 months. Participants who died between follow up time points will be assigned a QALY of 0 at the date of death and for remaining follow up points.

Incremental differences in QALYs will be calculated using linear mixed effects regression, adjusting for baseline utility scores, intervention group and site as fixed effects and allowing for clustering on therapy group^4^ , with participants in the usual care arm each assigned to a cluster of size 1 as described in section 3.1. The incremental difference and 95% confidence intervals will be calculated based on bootstrapped results. A fixed effects model will be used if the model fails to converge or if there is no evidence for clustering by therapist.

### 4.2 Discounting

We will apply a 3.5% annual discount rate for costs and outcomes to the 12 to 24 month follow up period in line with NICE guidance^8^.

## PRIMARY ANALYSIS

The principal economic analysis will be the **incremental cost per quality adjusted life year (QALY) gained of the APPLE-Tree intervention compared to the control at 24 months**, adjusting for baseline differences in utilities and costs.

All analyses will be carried out on an intention-to-treat basis, following methods outlined in the SAP, describing the groups as randomised based on all available outcome data.

### 5.1 Incremental cost-effectiveness ratio (ICER)

We will report mean incremental cost per QALY gained between the APPLE-Tree intervention and control arm at **24 months**. This analysis will use 2-stage bootstrap and bias corrected and accelerated (BCa) standard error to allow for the non-parametric assumption in the bootstrapping procedure. We will calculate the adjusted mean and, adjusted incremental (differences) in costs and QALYs between the intervention and control arm and 95% confidence intervals also using BCa approach (adjusting for baseline utilities, baseline costs and site as a fixed effect and with random effects as above).

If the mixed effects models fail to converge, or there is no evidence of clustering we will conduct an analysis using seemingly unrelated regression and bootstrapping with BCa to calculate the mean incremental adjusted costs and QALYs,

### 5.2 Cost effectiveness acceptability curve (CEAC) and cost-effectiveness plane (CEP)

The bootstrapped increments for costs and QALYs will be used to calculate the probability that the Apple-Tree intervention is cost-effective compared to the control arm for a range of cost-effectiveness thresholds for one QALY gained at 24 months and to generate the cost-effectiveness plane.

### 5.3 Missing data

Patterns of missing data in primary outcome measures and selected resource use data will be examined, following the principles set out in the statistical analysis plan (SAP). These analyses assume missing data is missing at random.

Multiple imputation (MI) will be used if >15 % of participants are missing resource use and/or EQ-5D-5L scores. Principles of MI described in the SAP will be followed, including the number of imputations will reflect the proportion of missingness.

Multiple imputation by chained equations (MICE) will be used to impute missing data and the ICER, CEAC and CEP will be reported based on imputed results using methods described by Leurent and colleagues^9^. We will use two-stage bootstrap of seemingly unrelated regression as described above.

### 5.4 Sensitivity analyses

We will conduct a series of sensitivity analyses to test the impact of changing key assumptions on the ICER.

We will vary the cost of the APPLE-Tree intervention, including the NHS band of the facilitator, the number of sessions attended (of the 10 offered) and the proportion of patients receiving the sessions at home (in person) and via video-call. We will also include a cost estimate for a case load model of costing.

The cost of private accommodation and out of pocket payments will be reported separately as they fall outside of the NHS and PSS perspective and will not be included in the main cost utility analysis.

## SECONDARY WITHIN-TRIAL ANALYSIS

As an alternative analysis, the incremental cost per unit change in NTB will be assessed only if there is a statistically significant difference in NTB in favour of the intervention in the primary analyses defined by the SAP and mean point estimate for the incremental costs is positive i.e. the intervention costs more. This analysis will include calculating a CEP and CEAC using the same methods set out above, If the intervention costs less and there is a statistically significant difference in NTB in favour of the intervention then there will be no need to conduct the analysis given that the intervention is dominant so cost-effective. If there is no significant effect, then adding an NTB secondary analysis will provide little additional information.

## REFERENCES

1 Poppe M, Duffy L, Marchant NL, *et al.* The APPLE Tree programme: Active Prevention in People at risk of dementia through Lifestyle, bEhaviour change and Technology to build REsiliEnce-randomised controlled trial. *Trials* 2022; **23**. DOI:10.1186/S13063-022-06557-6.

2 BNF (British National Formulary) | NICE. https://bnf.nice.org.uk/ (accessed Oct 4, 2024).

3 Unit Costs of Health and Social Care programme (2022 – 2027) | The new home for the Unit Costs of Health and Social Care report. https://www.pssru.ac.uk/unitcostsreport/ (accessed Oct 4, 2024).

4 Hunter RM, Baio G, Butt T, Morris S, Round J, Freemantle N. An Educational Review of the Statistical Issues in Analysing Utility Data for Cost-Utility Analysis. *Pharmacoeconomics* 2015; **33**: 355–66.

5 EQ-5D-5L | EuroQol. https://euroqol.org/information-and-support/euroqol-instruments/eq-5d-5l/ (accessed Oct 4, 2024).

6 Hernández Alava M, Pudney S, Wailoo A. FINAL REPORT Estimating the relationship between EQ-5D-5L and EQ-5D-3L: results from an English Population Study. 2 Acknowledgements. 2020.

7 Devlin NJ, Shah KK, Feng Y, Mulhern B, Van Hout B. Valuing health-related quality of life: An EQ-5D-5L value set for England-specific PROs focus on specific health. 2017. DOI:10.1002/hec.3564.

8 Overview | NICE health technology evaluations: the manual | Guidance | NICE. 2024. https://www.nice.org.uk/process/pmg36 (accessed Feb 21, 2025).

9 Leurent B, Gomes M, Faria R, Morris S, Grieve R, Carpenter JR. Sensitivity Analysis for Not-at-Random Missing Data in Trial-Based Cost-Effectiveness Analysis: A Tutorial. *Pharmacoeconomics* 2018; **36**: 889–901.

**Appendix 2: Figure 1 - Trial CONSORT diagram (also in** (1)**)**

**Appendix 2: Supplementary material table 1 - Baseline participant characteristics by arm**

|  | N | Control (n=372) | N | Intervention (n=374) |
| --- | --- | --- | --- | --- |
| Site: n (%) | 372 |  | 374 |  |
| North London |  | 22 (5·9%) |  | 22 (5·9%) |
| NELFT |  | 8 (2·2%) |  | 10 (2·7%) |
| Brighton |  | 31 (8·3%) |  | 32 (8·6%) |
| Barnet, Enfield & Haringey |  | 59 (15·9%) |  | 60 (16·0%) |
| Essex |  | 55 (14·8%) |  | 55 (14·7%) |
| Hounslow |  | 42 (11·3%) |  | 41 (11·0%) |
| Kent |  | 59 (15·9%) |  | 58 (15·5%) |
| Suffolk |  | 61 (16·4%) |  | 62 (16·6%) |
| Berkshire |  | 11 (3·0%) |  | 10 (2·7%) |
| Norfolk |  | 18 (4·8%) |  | 18 (4·8%) |
| Hertfordshire |  | 6 (1·6%) |  | 6 (1·6%) |
| Age (years): mean (SD) | 370 | 74·4 (7·2) | 372 | 74·3 (6·6) |
| Sex: n (%) | 371 |  | 372 |  |
| Male |  | 198 (53·4%) |  | 194 (52·2%) |
| Female |  | 173 (46·6%) |  | 177 (47·6%) |
| Other |  | 0 (0%) |  | 1 (0·3%) |
| Ethnicity: n (%) | 370 |  | 372 |  |
| White UK |  | 299 (80·8%) |  | 300 (80·6%) |
| White other |  | 32 (8·6%) |  | 25 (6·7%) |
| Asian |  | 27 (7·3%) |  | 23 (6·2%) |
| Black |  | 3 (0·8%) |  | 10 (2·7%) |
| Mixed |  | 4 (1·1%) |  | 12 (3·2%) |
| Arab |  | 2 (0·5%) |  | 1 (0·3%) |
| Other |  | 3 (0·8%) |  | 1 (0·3%) |
| First language: n (%) | 370 |  | 371 |  |
| English |  | 333 (90·0%) |  | 339 (91·4%) |
| Not English |  | 37 (10·0%) |  | 32 (8·6%) |
| Marital status: n (%) | 370 |  | 372 |  |
| Single |  | 22 (5·9%) |  | 24 (6·5%) |
| Married/civil partnership |  | 231 (62·4%) |  | 233 (62·6%) |
| Living with partner |  | 19 (5·1%) |  | 11 (3·0%) |
| Widowed |  | 51 (13·8%) |  | 57 (15·3%) |
| Divorced |  | 46 (12·4%) |  | 46 (12·4%) |
| Unable to specify |  | 1 (0·3%) |  | 1 (0·3%) |
| Highest level of education; n (%) | 370 |  | 372 |  |
| No Education |  | 1 (0·3%) |  | 1 (0·3%) |
| Primary |  | 4 (1·1%) |  | 6 (1·6%) |
| Secondary (e.g. O level; GCSE) |  | 81 (21·9%) |  | 86 (23·1%) |
| Further (e.g. A level; BTEC; NVQ) |  | 99 (26·8%) |  | 100 (26·9%) |
| Degree |  | 109 (29·5%) |  | 95 (25·5%) |
| Postgraduate |  | 67 (18·1%) |  | 80 (21·5%) |
| Other |  | 8 (2·2%) |  | 3 (0·8%) |
| Unable to specify |  | 1 (0·3%) |  | 1 (0·3%) |
| Employment: n (%) | 370 |  | 372 |  |
| Full time employment |  | 19 (5·1%) |  | 13 (3·5%) |
| Part time employment |  | 24 (6·5%) |  | 30 (8·1%) |
| Retired |  | 301 (81·4%) |  | 306 (82·3%) |
| Unemployed/unable to work |  | 9 (2·4%) |  | 8 (2·2%) |
| Other |  | 16 (4·3%) |  | 13 (3·5%) |
| Unable to specify |  | 1 (0·3%) |  | 2 (0·5%) |
| Living situation: n (%) | 370 |  | 372 |  |
| Live alone |  | 96 (25·9%) |  | 102 (27·4%) |
| Live with partner/relatives |  | 266 (71·9%) |  | 266 (71·5%) |
| Live with friends/other people |  | 3 (0·8%) |  | 3 (0·8%) |
| Other |  | 5 (1·4%) |  | 1 (0·3%) |
| Accommodation: n (%) | 370 |  | 372 |  |
| Council rented |  | 19 (5·1%) |  | 14 (3·8%) |
| Private rented |  | 19 (5·1%) |  | 21 (5·6%) |
| Own home |  | 327 (88·4%) |  | 326 (87·6%) |
| Supported living |  | 3 (0·8%) |  | 9 (2·4%) |
| Other |  | 2 (0·5%) |  | 2 (0·5%) |
| APOE genotype: n (%) | 331 |  | 339 |  |
| E2E2 |  | 1 (0·3%) |  | 0 (0%) |
| E2E3 |  | 35 (10·6%) |  | 35 (10·3%) |
| E2E4 |  | 5 (1·5%) |  | 5 (1·5%) |
| E3E3 |  | 206 (62·2%) |  | 206 (60·8%) |
| E3E4 |  | 78 (23·6%) |  | 86 (25·4%) |
| E4E4 |  | 6 (1·8%) |  | 7 (2·1%) |
| MCI/SCD: n (%) | 372 |  | 373 |  |
| Mild cognitive impairment |  | 155 (41·7%) |  | 153 (41·0%) |
| Subjective cognitive decline |  | 217 (58·3%) |  | 220 (59·0%) |

Abbreviations: APOE, Apolipoprotein E; NELFT, North East London NHS Foundation Trust; SD, standard deviation

**Appendix 3: Supplementary material table 2 - Descriptive statistics for community, primary and secondary health and social care resource use**

|  | Intervention (Apple-Tree) | | | Control | | |
| --- | --- | --- | --- | --- | --- | --- |
|  | total | n yes(%) | mean(SD)* | total | n yes(%) | mean(SD)* |
| NHS Direct - baseline | 179 | 18 (10.06%) | 1.44 (0.7) | 203 | 32 (8.72%) | 1.75 (1.92) |
| 12-months | 134 | 19 (14.18%) | 1.24 (0.63) | 134 | 14 (4.84%) | 1.36 (0.63) |
| 24-months | 116 | 19 (16.38%) | 1.11 (0.32) | 144 | 35 (13.16%) | 1.23 (0.6) |
| Emergency call-baseline | 179 | 12 (6.7%) | 1.25 (0.62) | 203 | 15 (4.09%) | 1.47 (0.74) |
| 12-months | 134 | 10 (7.46%) | 1.1 (0.32) | 134 | 11 (3.81%) | 1.09 (0.3) |
| 24-months | 116 | 9 (7.76%) | 1.22 (0.44) | 144 | 18 (6.77%) | 1.28 (0.57) |
| Paramedic only -baseline | 179 | 5 (2.79%) | 1.2 (0.45) | 203 | 3 (0.82%) | 1.0 (0.0) |
| 12-months | 134 | 7 (5.22%) | 1.14 (0.38) | 134 | 6 (2.08%) | 1.0 (0.0) |
| 24-months | 116 | 1 (0.42%) | 1.0 | 144 | 8 (3.01%) | 1.0 (0) |
| Paramedic and ambulance - baseline | 179 | 14 (7.82%) | 1.23 (0.6) | 203 | 23 (6.27%) | 1.17 (0.39) |
| 12-months | 134 | 11 (8.21%) | 1.27 (0.65) | 134 | 12 (4.15%) | 1.08 (0.29) |
| 24-months | 116 | 8 (6.9%) | 1.25 (0.46) | 144 | 21 (7.89%) | 1.33 (0.66) |
| A&E only-baseline | 179 | 28 (15.64%) | 1.07 (0.26) | 203 | 34 (9.26%) | 1.29 (0.58) |
| 12-months | 134 | 26 (19.4%) | 1.36 (0.64) | 134 | 23 (7.96%) | 1.18 (0.39) |
| 24-months | 116 | 17 (14.66%) | 1.24 (0.44) | 144 | 26 (9.77%) | 1.42 (0.58) |
| Outpatient: general medicine-baseline | 179 | 105 (58.66%) | 2.3 (2.71) | 203 | 116 (31.61%) | 2.26 (3.18) |
| 12-months | 134 | 84 (62.69%) | 2.41 (2.35) | 134 | 76 (26.3%) | 2.27 (1.74) |
| 24-months | 116 | 65 (56.03%) | 2.86 (2.34) | 144 | 91 (34.21%) | 3.87 (12.66) |
| Outpatient: psychiatric-baseline | 179 | 3 (1.68%) | 7.33 (9.24) | 203 | 5 (1.36%) | 1.8 (0.84) |
| 12-months | 134 | 4 (2.99%) | 1.25 (0.5) | 134 | 2 (0.69%) | 1.5 (0.71) |
| 24-months | 116 | 4 (3.45%) | 3.25 (2.63) | 144 | 2 (0.75%) | 2.0 (1.41) |
| Memory service-baseline | 179 | 24 (13.41%) | 1.5 (0.88) | 203 | 19 (5.18%) | 1.53 (0.77) |
| 12-months | 134 | 17 (12.69%) | 1.12 (0.33) | 134 | 13 (4.5%) | 1.58 (0.9) |
| 24-months | 116 | 12 (10.34%) | 2.25 (1.66) | 144 | 10 (3.76%) | 1.9 (2.85) |
| Outpatient: physiotherapy-baseline | 179 | 22 (12.29%) | 2.95 (3.66) | 203 | 20 (5.45%) | 4.9 (5.76) |
| 12-months | 134 | 16 (11.94%) | 2.62 (2.25) | 134 | 7 (2.42%) | 6.0 (11.76) |
| 24-months | 116 | 18 (15.52%) | 2.82 (2.53) | 144 | 23 (8.65%) | 3.7 (3.01) |
| Outpatient: OT-baseline | 179 | 3 (1.68%) | 3.33 (4.04) | 203 | 0 | 0 |
| 12-months | 134 | 3 (2.24%) | 1.33 (0.58) | 134 | 4 (1.38%) | 1.75 (1.5) |
| 24-months | 116 | 3 (1.26%) | 1.0 (0) | 144 | 1 (0.38%) | 1.0 |
| Outpatient: SLT - baseline | 179 | 3 (0.82%) | 1.0 (0.0) | 203 | 0 | 0 |
| 12-months | 134 | 2 (0.69%) | 1.0 (0.0) | 134 | 0 | 0 |
| 24-months | 116 | 3 (1.26%) | 1.0 (0.0) | 144 | 1 (0.38%) | 1.0 (0.0) |
| Day care - baseline | 179 | 65 (36.31%) | 2.21 (2.1) | 203 | 102 (27.79%) | 2.25 (3.34) |
| 12-months | 134 | 64 (47.76%) | 2.2 (1.99) | 134 | 51 (17.65%) | 1.65 (1.29) |
| 24-months | 116 | 60 (51.72%) | 1.78 (1.34) | 144 | 76 (28.57%) | 2.28 (2.15) |
| GP: clinic - baseline | 244 | 134 (54.92%) | 1.57 (1.16) | 228 | 124 (33.79%) | 1.52 (0.99) |
| 12-months | 181 | 131 (72.38%) | 1.74 (1.31) | 192 | 150 (51.9%) | 1.79 (1.3) |
| 24-months | 175 | 145 (82.86%) | 2.04 (1.84) | 186 | 154 (57.89%) | 2.08 (1.53) |
| GP: video - baseline | 244 | 8 (3.28%) | 1.12 (0.35) | 228 | 7 (1.91%) | 1.14 (0.38) |
| 12-months | 181 | 8 (2.77%) | 1.14 (0.38) | 192 | 1 (0.35%) | 1.0 |
| 24-months | 175 | 2 (0.84%) | 1.0 (0) | 186 | 1 (0.38%) | 3.0 |
| GP: phone - baseline | 244 | 181 (74.18%) | 2.26 (3.33) | 228 | 155 (42.23%) | 2.21 (1.86) |
| 12-months | 181 | 102 (56.35%) | 2.06 (1.4) | 192 | 98 (33.91%) | 2.01 (1.86) |
| 24-months | 175 | 74 (42.29%) | 2.11 (1.81) | 186 | 85 (31.95%) | 1.8 (1.04) |
| GP: home - baseline | 244 | 4 (1.64%) | 1.0 (0.0) | 228 | 5 (1.36%) | 1.6 (0.89) |
| 12-months | 181 | 5 (2.76%) | 1.25 (0.5) | 192 | 5 (1.73%) | 6.0 (8.46) |
| PCN: clinic - baseline | 162 | 149 (91.98%) | 1.69 (1.53) | 154 | 134 (36.51%) | 1.7 (1.33) |
| 12-months | 115 | 111 (96.52%) | 2.12 (3.14) | 137 | 128 (44.29%) | 1.81 (2.05) |
| 24-months | 132 | 131 (99.24%) | 1.82 (1.4) | 149 | 144 (54.14%) | 2.19 (2.5) |
| PCN: phone - baseline | 162 | 17 (10.49%) | 1.47 (1.28) | 154 | 30 (8.17%) | 1.4 (1.0) |
| 12-months | 115 | 9 (7.83%) | 1.11 (0.33) | 137 | 15 (5.19%) | 1.46 (0.97) |
| 24-months | 132 | 8 (6.06%) | 1.38 (0.52) | 149 | 6 (2.26%) | 1.17 (0.41) |
| CN: clinic - baseline | 28 | 26 (92.86%) | 3.04 (6.36) | 27 | 25 (6.81%) | 4.2 (9.74) |
| 12-months | 11 | 7 (63.64%) | 1.0 (0.0) | 9 | 6 (2.08%) | 22.33 (42.71) |
| 24-months | 6 | 4 (66.67%) | 2.25 (1.5) | 11 | 7 (2.63%) | 20.0 (44.22) |
| HCA: clinic - baseline | 17 | 14 (82.35%) | 1.29 (0.47) | 15 | 13 (3.54%) | 1.31 (0.63) |
| 12-months | 7 | 6 (85.71%) | 1.2 (0.45) | 7 | 6 (2.08%) | 2.17 (1.33) |
| 24-months | 11 | 9 (81.82%) | 1.56 (1.01) | 11 | 11 (4.14%) | 2.0 (1.26) |
| HCA: phone - baseline | 17 | 5 (29.41%) | 1.8 (1.79) | 15 | 2 (0.54%) | 1.0 (0.0) |
| 12-months | 7 | 1 (14.29%) | 1 | 7 | 0 | 0 |
| 24-months | 11 | 1 (9.09%) | 1 | 11 | 0 | 0 |
| CMHT: clinic - baseline | 10 | 4 (40.0%) | 2.5 (1.29) | 15 | 6 (1.63%) | 1.67 (0.82) |
| 12-months | 6 | 2 (33.33%) | 1.0 (0.0) | 7 | 7 (100%) | 3.43 (5.13) |
| 24-months | 6 | 6 (100.0%) | 4.33 (7.69) | 7 | 5 (71.43%) | 12.4 (12.1) |
| CMHT: phone - baseline | 10 | 4 (40.0%) | 1.0 (0.0) | 15 | 9 (2.45%) | 3.44 (3.78) |
| 12-months | 6 | 3 (50.0%) | 1.67 (0.58) | 7 | 1 (14.29%) | 8 |
| 24-months | 6 | 2 (33.33%) | 2.0 (1.41) | 7 | 2 (0.75%) | 1.5 (0.71) |
| Community: OT clinic - baseline | 6 | 3 (50.0%) | 2.0 (1.73) | 5 | 3 (0.82%) | 1.0 (0.0) |
| 12-months | 7 | 7 (100%) | 2.57 (3.31) | 7 | 1 (14.29) | 1.0 |
| 24-months | 5 | 4 (80.0%) | 1.75 (0.96) | 6 | 3 (1.13%) | 3.67 (0.58) |
| Community: physiotherapist - baseline | 40 | 29 (72.5%) | 3.62 (5.22) | 32 | 29 (7.9%) | 1.76 (1.62) |
| 12-months | 37 | 34 (91.89%) | 2.53 (2.51) | 21 | 21 (7.27%) | 2.43 (2.27) |
| 24-months | 30 | 28 (93.33%) | 3.86 (5.28) | 33 | 28 (10.53%) | 2.07 (1.7) |
| Community: physiotherapist(phone) - baseline | 40 | 7 (17.5%) | 1.14 (0.38) | 32 | 5 (1.36%) | 2.6 (2.3) |
| 12-months | 37 | 5 (13.51%) | 1.4 (0.89) | 21 | 2 (0.75%) | 1.0 (0.0) |
| 24-months | 30 | 2 (6.67%) | 1.5 (0.71) | 33 | 2 (0.75%) | 1.5 (0.71) |
| Optitian - baseline | 322 | 110 (34.16%) | 1.29 (0.94) | 311 | 133 (36.24%) | 1.18 (0.55) |
| 12-months | 228 | 114 (50.0%) | 1.2 (0.8) | 234 | 111 (38.41%) | 1.17 (0.59) |
| 24-months | 208 | 108 (51.92%) | 1.27 (0.62) | 231 | 119 (44.74%) | 1.24 (0.71) |
| SW - baseline | 322 | 4 (1.24%) | 1.33 (0.58) | 311 | 3 (0.82%) | 1.67 (1.15) |
| 12-months | 228 | 4 (1.75%) | 2.62 (1.97) | 234 | 3 (1.04%) | 1.33 (0.58) |
| 24-months | 208 | 3 (1.44%) | 1.0 (0) | 231 | 2 (0.87%) | 1.0 (0) |
| Podiatrist/chiropodist - baseline | 322 | 55 (17.08%) | 2.02 (3.3) | 311 | 58 (15.8%) | 2.14 (3.4) |
| 12-months | 228 | 43 (18.86%) | 2.52 (3.59) | 234 | 53 (18.34%) | 2.1 (1.36) |
| 24-months | 208 | 39 (18.75%) | 3.1 (4.67) | 231 | 54 (20.3%) | 2.5 (2.11) |
| Counselling - baseline | 322 | 14 (4.35%) | 6.79 (5.94) | 311 | 14 (3.81%) | 5.07 (3.71) |
| 12-months | 228 | 16 (7.02%) | 4.67 (3.11) | 234 | 5 (1.73%) | 2.6 (2.07) |
| 24-months | 208 | 8 (3.85%) | 5.12 (7.79) | 231 | 3 (1.13%) | 4.67 (0.58) |
| Pharmacist - baseline, | 322 | 25 (7.76%) | 2.21 (3.54) | 311 | 24 (6.54%) | 1.21 (0.41) |
| 12-months | 228 | 21 (9.21%) | 2.0 (2.61) | 234 | 24 (8.3%) | 2.08 (2.45) |
| 24-months | 208 | 32 (15.38%) | 1.47 (0.84) | 231 | 38 (14.29%) | 1.21 (0.47) |
| Group therapy - baseline | 322 | 8 (2.48%) | 19.75 (23.73) | 311 | 1 (0.32%) | 78 |
| 12-months | 228 | 2 (0.88%) | 1.0 (0.0) | 234 | 4 (1.38%) | 2.5 (2.38) |
| 24-months | 208 | 2 (0.96%) | 16.0 (5.66) | 231 | 5 (1.88%) | 6.2 (4.66) |
| Home Help (hours per week) - baseline | 322 | 10 (3.11%) | 87.9 (156.29) | 311 | 9 (2.45%) | 54.0 (74.36) |
| 12-weeks | 228 | 3 (1.32%) | 67.0 (100.5) | 234 | 3 (1.04%) | 62.0 (91.85) |
| 24-weeks | 208 | 6 (2.88%) | 81.0 (92.68) | 231 | 9 (3.38%) | 32.25 (17.25) |
| Community: SLT - baseline | 322 | 1 (0.31%) | 3.0 | 311 | 0 |  |
| 12-weeks | 228 | 2 (0.88%) | 1.5 (0.71) | 234 | 1 (0.43%) | 1.0 |
| 24-weeks | 208 | 4 (1.92%) | 1.0 (0.0) | 231 | 2 (0.75%) | 4.0 (4.24) |

* Non-zeros only

NHS = National Health Service; A&E = Accident and emergency; OT = Occupational therapist; SLT = Speech and language therapist; GP = General practitioner; PCN = Primary care nurse; CN = Community nurse; HCA = Health care assistant; CMHT = Community mental health team; SW = Social worker.

**Appendix 4: Supplementary material table 3 - Descriptive statistics for inpatient resource use**

|  | Intervention (Apple-Tree) | | | Control | | |
| --- | --- | --- | --- | --- | --- | --- |
|  | total | n yes(%) | mean(SD)* | total | n yes(%) | mean(SD)* |
| number unplanned admissions: baseline | 366 | 15 (0.04%) | 1.07 (0.26) | 367 | 24 (0.07%) | 1.29 (0.91) |
| 12-months | 289 | 10 (0.03%) | 1.2 (0.63) | 289 | 10 (0.03%) | 1.1 (0.32) |
| 24-months | 239 | 11 (0.05%) | 1.09 (0.3) | 266 | 13 (0.05%) | 1.23 (0.6) |
| number unplanned nights: baseline | 366 | 15 (0.04%) | 5.87 (6.89) | 367 | 24 (0.07%) | 7.29 (15.83) |
| 12-months | 289 | 10 (0.03%) | 8.5 (16.83) | 289 | 10 (0.03%) | 6.7 (9.63) |
| 24-months | 239 | 11 (0.05%) | 6.64 (14.49) | 266 | 13 (0.05%) | 5.08 (4.57) |
| number planned admissions: baseline | 366 | 10 (0.03%) | 1.1 (0.32) | 367 | 5 (0.01%) | 1.2 (0.45) |
| 12-months | 289 | 7 (0.02%) | 1.0 (0.0) | 289 | 7 (0.02%) | 1.14 (0.38) |
| 24-months | 239 | 7 (0.03%) | 1.0 (0.0) | 266 | 8 (0.03%) | 1.25 (0.46) |
| number planned nights: baseline | 366 | 10 (0.03%) | 2.3 (3.09) | 367 | 5 (0.01%) | 5.2 (4.92) |
| 12-months | 289 | 7 (0.02%) | 2.86 (2.91) | 289 | 7 (0.02%) | 2.43 (1.9) |
| 24-months | 239 | 7 (0.03%) | 1.57 (0.79) | 266 | 8 (0.03%) | 2.62 (2.07) |
